# Midlife Neuroinflammatory Dynamics: The Emergence of Alzheimer’s Disease Neuroimmune Profile in the Female Human Brain

**DOI:** 10.1002/alz.093151

**Published:** 2025-01-03

**Authors:** Hannah Van Rossum, Yuan Shang, Roberta Diaz Brinton

**Affiliations:** ^1^ University of Arizona, Tucson, AZ USA

## Abstract

**Background:**

Inflammation plays a pivotal role in driving the development and progression of Alzheimer’s disease (AD) in the human brain, offering a promising avenue for therapeutic intervention. However, the initiation phase of inflammation and its potential sex differences remain elusive. In this study, we aim to provide translational validity to our preclinical findings by testing two hypotheses: 1) the inflammatory profile of late‐onset AD (LOAD) is initiated and detectable during midlife aging, and 2) sex differences manifest in the brain by midlife.

**Method:**

Human frozen tissue samples were procured from the NIH NeuroBioBank (University of Maryland, University of Miami, and Mt. Sinai). We matched samples from 40 brain donors for sex and age, encompassing tissues from the hippocampus, hypothalamus, and corpus callosum. APOE genotyping determined APOE e4 carrier status. The study classified samples into three groups: healthy aging 40‐49 years old (young controls), healthy aging 50‐59 years old (old controls), and AD aged 60‐80 years old (AD). Exploratory analyses utilized Nanostring’s Neuroinflammation Panel, with subsequent replication through deep in‐depth characterization by bulk‐seq at Vanderbilt University Medical Center.

**Result:**

Principal components analysis indicated that transcriptomic profiles are influenced by cofactors such as tissue, sex, and disease state. Unbiased pathway analysis identified the top 5 upregulated pathways in AD compared to healthy aging controls, all associated with the immune system and inflammation. Conversely, the top 5 down‐regulated pathways were linked to synaptic function. Targeted pathway analysis, including peripheral immune cell regulation, interferon, and microglial activation, revealed significant sex and APOE e4 carrier differences in all three brain regions.

**Conclusion:**

This comprehensive characterization provides compelling evidence of sex differences in neuroinflammation emerging during midlife. Notably, the midlife female brain exhibits a profile reminiscent of the inflammatory signature observed in the AD brain. The study presents an opportunity to identify distinct profiles emerging decades before AD diagnosis, and to inform precision medicine interventions that can be implemented to prevent or delay a key driver of AD pathology.

